# Tachycardia Termination Without Global Propagation: Is the Pacing Site In or Out?

**DOI:** 10.19102/icrm.2025.16123

**Published:** 2025-12-15

**Authors:** Ozcan Ozeke, Dursun Aras, Serkan Topaloglu

**Affiliations:** 1 Department of Cardiology, University of Health Sciences, Ankara Bilkent City Hospital, Ankara, Turkey; 2 Department of Cardiology, İstanbul Medipol University, Istanbul, Turkey

**Keywords:** Non-global capture, tachycardia termination without global propagation, ventricular tachycardia

## Abstract

Ventricular tachycardia termination by pacing with non-global capture is a specific criterion for identifying a critical component of the re-entrant circuit, regardless of whether concealed entrainment can be demonstrated at that site. It is usually observed almost by chance, but it can also be intentionally demonstrated by introducing a single extrastimulus during tachycardia.

## Case presentation

**Figure 1:**
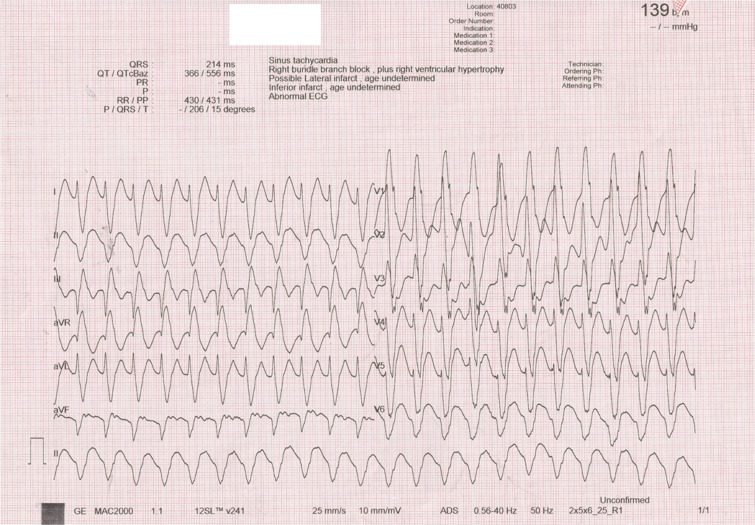
A 12-lead electrocardiogram showing the clinical ventricular tachycardia.

**Figure 2:**
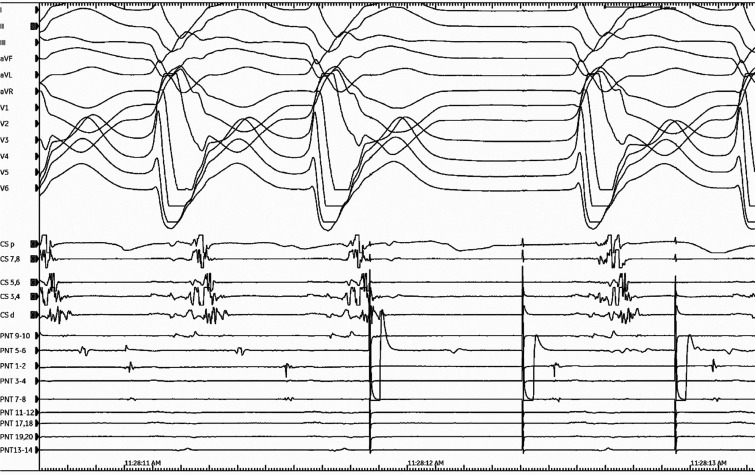
Termination without global propagation by the first stimulus of a pacing train from the PentaRay^®^ catheter (PNT) 5-6 is seen.

A 72-year-old man was referred for catheter ablation of incessant ventricular tachycardia (VT). He had experienced a myocardial infarction 12 years earlier and had previously undergone coronary bypass and mitral valve replacement surgery. An electrocardiogram revealed sustained monomorphic VT characterized by a QRS morphology with a right bundle branch block pattern and a superior axis; the tachycardia cycle length was 430 ms **(Figure 1)**. The clinical VT was spontaneously induced and was hemodynamically tolerated and mapped **(Video 1)**. During the tachycardia, a series of single ventricular extrastimuli was delivered from the putative diastolic potential region. Based on the response to ventricular entrainment attempts **(Figure 2)**, we questioned whether the pacing site was in or out of the re-entrant circuit.

## Discussion

In the setting of structural heart disease, VT is typically associated with a re-entrant mechanism.^1–4^ Successful catheter ablation of VT depends on identifying the critical tissues that sustain the arrhythmia.^5^ Four mapping techniques consistently play a role in successful VT ablation: activation mapping, entrainment mapping, pace mapping, and substrate mapping. Entrainment mapping, or continuous resetting of a re-entrant tachycardia, provides a powerful tool for distinguishing between focal and re-entrant VT, as well as identifying critical circuit components such as the entrance, critical isthmus (CI), and exit sites.^1,6–12^ The detection of the ideal site for ablation within a central CI is a mid-diastolic potential during VT that exhibits concealed fusion with a post-pacing interval equal to the tachycardia cycle length.^13^ Thus, entrainment mapping helps to identify the CI, but it is not always feasible and, in some cases, terminates VT,^14^ as occurred in the current tracing. Indeed, local capture of the CI without global ventricular capture (non-global capture [NGC]) can terminate VT by resulting in a bidirectional block (orthodromic or non-orthodromic capture) within the CI.^15–18^ However, termination might also occur due to a rate-dependent block in the slow-conduction zone during pacing,^4,19,20^ and catheter contact at a critical endocardial site can interrupt postinfarction VT.^21^ The stimulus–QRS (S–QRS) interval was significantly shorter at sites where mechanical trauma affected the re-entrant circuit compared with sites having concealed entrainment.^21^ Therefore, if an arrhythmia terminates during overdrive pacing but several stimuli that should capture do not, then the termination might have been spontaneous or caused by mechanical trauma; such events do not carry the same implications as termination by NGC. Furthermore, all these pacing attempts rely on consistent capture of tissue with each stimulus. While “capture” refers to whether the pacing stimulus depolarizes the local myocardium, “global capture” means that the entire QRS complex (ie, the full ventricular myocardium) is activated by the pacing stimulus and the morphology differs from the VT QRS. On the contrary, NGC (also termed “concealed entrainment”) means that only a part of the VT circuit or surrounding myocardium is depolarized without altering the overall QRS morphology during VT, supporting CI involvement.^15,18^ VT termination by pacing with NGC is a specific criterion for identifying a critical component of the re-entrant circuit, regardless of whether concealed entrainment can be demonstrated at that site.^15,16^ It is usually observed almost by chance, but it can also be intentionally demonstrated by introducing a single extrastimulus during tachycardia.

**Figure 3:**
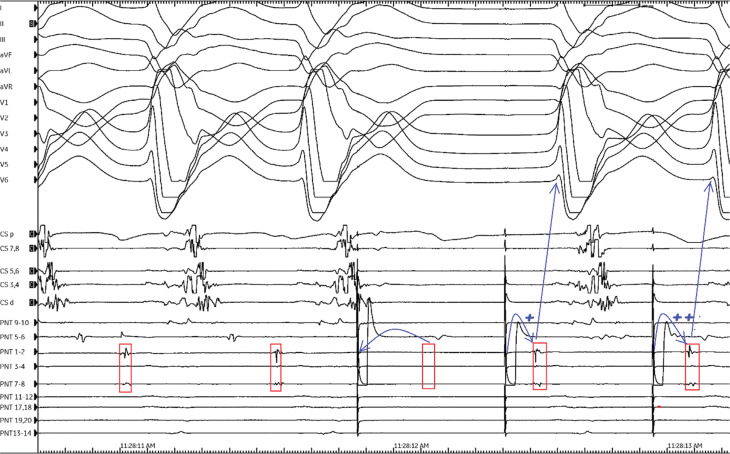
Illustration corresponding to **Figure 2**. Local electrograms on PentaRay^®^ 1-2 and 7-8 (red rectangles) confirm stimulus-induced capture (blank red rectangle). Conduction delay is evident by gradual stimulus–QRS prolongation after tachycardia termination by non-global capture (blue plus signs).

In the current tracing, VT was terminated with NGC by a single extrastimulus at this site; however, concealed entrainment could not be assessed due to the termination of the VT. In general, amplifier saturation from pacing typically obscures the local electrogram (EGM) on the pacing electrodes at the time of pacing, as seen in **Figure 2** (5-6 with PentaRay^®^ [JNJ Medtech, New Brunswick, NJ, USA]); thus, a careful examination of adjacent electrode recordings (PentaRay^®^ 1-2 and 7-8, **Figure 3**) is important to confirm evidence of local capture. Examination of the adjacent electrode recording (red rectangles in **Figure 3**) confirms that the similarly timed component was captured just downstream of the stimulus, as it is no longer observed at the expected interval (red blank rectangle in **Figure 3** indicates absent EGM).^14^

Following termination of VT, stimuli directly captured the local CI myocardium, probably including the exit site, as confirmed by the QRS morphology matching that of the VT.^19^ The S–QRS interval is a well-known indicator of the conduction time from the pacing site to the exit VT.^22^ If it is prolonged and furthermore reveals a conduction delay in the isthmus after VT termination by NGC, these support the NGC location as a critical position in the re-entrant circuit.^15^ In the current tracing, there was also evidence of a conduction delay (**Figure 3**, note the blue “plus sign”), with gradual prolongation of the S–QRS interval following tachycardia termination by NGC. Combining all these observations, ablation at this site rendered the clinical VT non-inducible.

## Supporting information

Supplementary Video 1: Activation mapping shows the re-entry circuit at the inferior left ventricle.
